# Study protocol of the CORRECT multicenter trial: the efficacy of blended cognitive behavioral therapy for reducing psychological distress in colorectal cancer survivors

**DOI:** 10.1186/s12885-018-4645-6

**Published:** 2018-07-18

**Authors:** L. Leermakers, S. Döking, B. Thewes, A. M. J. Braamse, M. F. M. Gielissen, J. H. W. de Wilt, E. H. Collette, J. Dekker, J. B. Prins

**Affiliations:** 1 Radboud Institute for Health Sciences, Department of Medical Psychology Radboud University Medical Center, (840), P.O. Box 9101, 6500 HB Nijmegen, The Netherlands; 2 Department of Medical Psychology, Amsterdam UMC, location AMC, P.O. Box 22660, 1100 DD Amsterdam, The Netherlands; 3 Radboud Institute for Health Sciences, Department of primary and community care, Radboud University Medical Center, P.O. Box 9101, 6500 HB Nijmegen, The Netherlands; 4 Siza (disability service) Arnhem, P.O. Box 532, 6800 AM Arnhem, The Netherlands; 5 Radboud Institute for Health Sciences, Department of Surgery, Radboud university medical center, (725), P.O. Box 9101, 6500 HB Nijmegen, The Netherlands; 6 Department of Medical Psychology, Amsterdam UMC, location VUmc, P.O. Box 7057, 1007 MB Amsterdam, The Netherlands; 7 Department of Rehabilitation Medicine, Amsterdam UMC, location VUmc, P.O. Box 7057, 1007 MB Amsterdam, The Netherlands; 8 Department of Psychiatry, Amsterdam UMC, location VUmc, P.O. Box 7057, 1007 MB Amsterdam, The Netherlands

**Keywords:** Colorectal cancer survivors, Psychological distress, Blended therapy, Cognitive behavior therapy, Quality of life, Randomized controlled trial

## Abstract

**Background:**

Approximately one third of the colorectal cancer survivors (CRCS) experience high levels of psychological distress. Common concerns experienced by CRCS include distress related to physical problems, anxiety, fear of cancer recurrence (FCR) and depressive symptoms. However, psychological interventions for distressed CRCS are scarce. Therefore, a blended therapy was developed, combining face-to-face cognitive behavioral therapy (CBT) with online self-management activities and telephone consultations. The aim of the study is to evaluate the efficacy and cost-effectiveness of this blended therapy in reducing psychological distress in CRCS.

**Methods/design:**

The CORRECT study is a two-arm multicenter randomized controlled trial (RCT). A sample of 160 highly distressed CRCS (a score on the Distress Thermometer of 5 or higher) will be recruited from several hospitals in the Netherlands. CRCS will be randomized to either the intervention condition (blended CBT) or the control condition (care as usual). The blended therapy covers approximately 14 weeks and combines five face-to-face sessions and three telephone consultations with a psychologist, with access to an interactive self-management website. It includes three modules which are individually-tailored to patient concerns and aimed at decreasing: 1) distress caused by physical consequences of CRC, 2) anxiety and FCR, 3) depressive symptoms. Patients can choose between the optional modules. The primary outcome is general distress (Brief Symptom Inventory-18). Secondary outcomes are quality of life and general psychological wellbeing. Assessments will take place at baseline prior to randomization, after 4 and 7 months.

**Discussion:**

Blended CBT is an innovative and promising approach for providing tailored supportive care to reduce high distress in CRCS. If the intervention proves to be effective, an evidence-based intervention will become available for implementation in clinical practice.

**Trial registration:**

This trial is registered in the Netherlands Trial Register (NTR6025) on August 3, 2016.

## Background

Colorectal cancer (CRC) is one of the most frequently diagnosed cancers, with over 3.5 million survivors worldwide [1]. Although the majority of the colorectal cancer survivors (CRCS) are resilient and eventually adjust well, a significant proportion of CRCS experience on-going high levels of chronic distress [2]. The National Comprehensive Cancer Network (NCCN) defines distress as “a multi-factorial unpleasant emotional experience of a psychological (cognitive, behavioral, emotional), social and/or spiritual nature that may interfere with the ability to cope effectively with cancer, its physical symptoms and its treatment. Distress extends along a continuum ranging from common normal feelings of vulnerability, sadness and fears to problems that can become disabling such as depression, anxiety, panic, social isolation and existential and spiritual crisis” [3]. Distress occurs in approximately one third of CRCS [4, 5]. Distress is an unfavorable outcome in itself and a known risk factor for a poor outcome following a cancer diagnosis in the physical, mental and social domains of quality of live (QoL) [6, 7].

The problems related to CRC which underlie distress are very broad. Most previous research has investigated the role of physical, emotional or social problems during the phase of survivorship. A systematic review found that long-term CRCS have good overall QoL [8]. However, the majority of CRCS may still experience problems that can adversely impact upon their daily life. Frequently experienced adverse effects of CRC and its treatment are fatigue [9, 10], pain [11], neuropathy [12], poor body image [13] and gastrointestinal problems [13–15]. A substantial amount of patients will have a permanent stoma after treatment for CRC and may experience ostomy-related problems including gas, constipation, change in clothing, travel difficulties, feeling tired, depressive feelings, and worry about odours and noises [16]. However, there is inconsistent evidence about whether or not there are differences in QoL amongst CRCS with and without a stoma [16, 17]. Sexual dysfunction after CRC treatment is a problem varying between 5 and 88% for men and approximately 50% of the women reported that problem [18].

In addition to distress caused by physical problems, anxiety and depressive symptoms are two major concerns of CRCS. The prevalence of anxiety and depressive symptoms amongst CRCS varies between studies. The reported prevalence of mild to moderate depressive symptoms among CRCS (0–6 years after diagnosis) ranges from 8 to 57% [9, 19–21]. Prevalence rates of mild anxiety in CRCS (0–6 years after diagnosis) vary between 14 and 83% [9, 20, 21]. Moderate levels of anxiety have been reported in 6–68% of CRCS [20, 21]. A specific cancer related fear is the fear of cancer recurrence (FCR), defined as the “fear, worry, or concern relating to the possibility that cancer will come back or progress” [22]. Low to moderate levels of fear can be adaptive, and can motivate appropriate health behavior and surveillance, however moderate to high levels of FCR can have a negative impact on mood, daily functioning and QoL [22]. In a large sample of 10,969 CRCS, 50% of respondents reported fear of their cancer returning [23]. Custers and colleagues [24] found that 38% of the CRCS (*N* = 76) experienced high levels of FCR above a clinically validated cut-off. These high levels of FCR were associated with higher levels of distress, post-traumatic stress and lower QoL. A systematic review of interventions for distress in cancer patients has shown that psychological interventions have small to medium effects on distress levels in cancer patients whereas studies that included specifically participants with high distress showed larger effect sizes [25]. However, most research on distress and psychosocial interventions has been conducted with mixed cancer survivors or breast cancer patients [25]. Due to the prevalence of CRC-related distress, specific physical problems associated with CRC and growing numbers of CRCS, providing interventions for distress in CRCS is of increasing importance.

Relatively few studies have explored the effectiveness of psychological interventions designed to improve emotional outcomes for CRCS. A recent systematic review on psychosocial interventions for CRC patients of all disease stages identified 14 randomized controlled trials RCTs [26]. Only three of these RCTs proved to be effective for different mental health outcomes. These three interventions investigated emotional expression, a progressive muscle relaxation training, and an intervention to enhance self-efficacy. Besides the RCTs described in this systematic review, four other studies were found investigating an intervention specifically for CRCS. Lepore and colleagues [27] tested in a randomized trial whether a home-based expressive writing intervention improved QoL in patients with CRC. The intervention was however not effective. Jefford and colleagues [28] developed an intervention (SurvivorCare) which was nurse-led and consisted of educational materials, needs assessment, survivorship care plan, end-of-treatment session and three follow-up telephone calls. The addition of SurvivorCare to usual care showed no beneficial effect. White and colleagues [29] investigated the effect of a volunteer-delivered telephone-based intervention on reducing anxiety and depression among patients recently diagnosed with CRC. Results indicated no change in depressive symptoms, although there was a reduction in anxiety. Hawkes and colleagues [30] tested another telephone-based intervention which was provided by health coaches and aimed at health behavior change. This intervention improved psychosocial outcomes and QoL, but there was no effect on distress.

To summarize, previous studies on psychological interventions for CRCS are inconsistent with most studies failing to demonstrate a positive effect of the intervention. Most existing studies did not select patients based on distress level [26–29] and therefore might have failed to identify those who might benefit the most from psychological interventions [25]. Furthermore, interventions were either nurse-led [26, 28, 29] or telephone-based [29, 30] which may be less sufficient to improve psychological outcomes compared to psychologist-led interventions. Finally, most intervention studies to date included either only short-term CRCS (< 1 year) or patients who were still during medical treatment [26, 28, 29]. Therefore, treatment efficacy for long-term CRCS is still unclear.

Due to the paucity of evidence-based interventions to reduce psychological distress in CRCS, and the limitations of existing intervention studies, there is an urgent need to develop and evaluate a cost-effective and easily accessible psychological intervention for CRCS. The CORRECT (COloRectal canceR distrEss reduCTion) intervention is a blended therapy to reduce psychological distress amongst CRCS which has been specifically developed to address this need. It is called blended therapy because it is a combination of face-to-face (F2F) cognitive behavioral therapy (CBT), interactive self-management activities at a secure website and telephone consultations. Blended therapy is an innovative and promising approach to psychological care delivery. It reduces therapist workload, and is known to lead to better outcomes and reduced patient dropout compared with internet-only interventions [31–34]. By adding online activities to F2F psychological therapy, patients access treatment at home at their convenience. These online activities consist of homework assignments. Towards the end of the intervention period, self-management is increased through the use of the interactive website. In this way patients take charge of their own health and learn to cope more independently with future challenges. Furthermore, blended therapy provided in the CORRECT intervention is tailored to the needs of each individual. As we know, the CRC population is diverse and includes survivors with a variety of different characteristics and treatments therefore the physical and psychosocial consequences vary between individuals. Distress can be seen as a multi-factorial cluster concept. Despite individual variations in symptom profiles, based on previous research, we know that the most common symptoms in CRCS are distress related to physical consequences, anxiety and depressive symptoms. Therefore, in the present study the intervention is tailored according to individual needs in three optional modules: 1) distress due to physical consequences of CRC, 2) anxiety and FCR, and 3) depressive symptoms. The primary objective of the CORRECT study is to evaluate the efficacy and cost-effectiveness of the CORRECT intervention in decreasing psychological distress in CRCS. A secondary aim is to investigate the usage of online activities at the secure website and how online usage is associated with distress reduction.

## Methods/design

The design, and evaluation of this intervention are in accordance with guidelines of conducting Internet intervention research [35], the CONSORT 2010 statement for parallel group randomized trials [36], and for eHealth interventions [37]. The Medical Ethics Committee of the Radboud university medical center (CMO Arnhem-Nijmegen) (NL55018.091.15) and relevant hospital and institutional human research ethics committees granted ethical approval. The current study is registered in the Netherlands National Trial Register (NTR6025).

### Study design

**Fig. 1:**
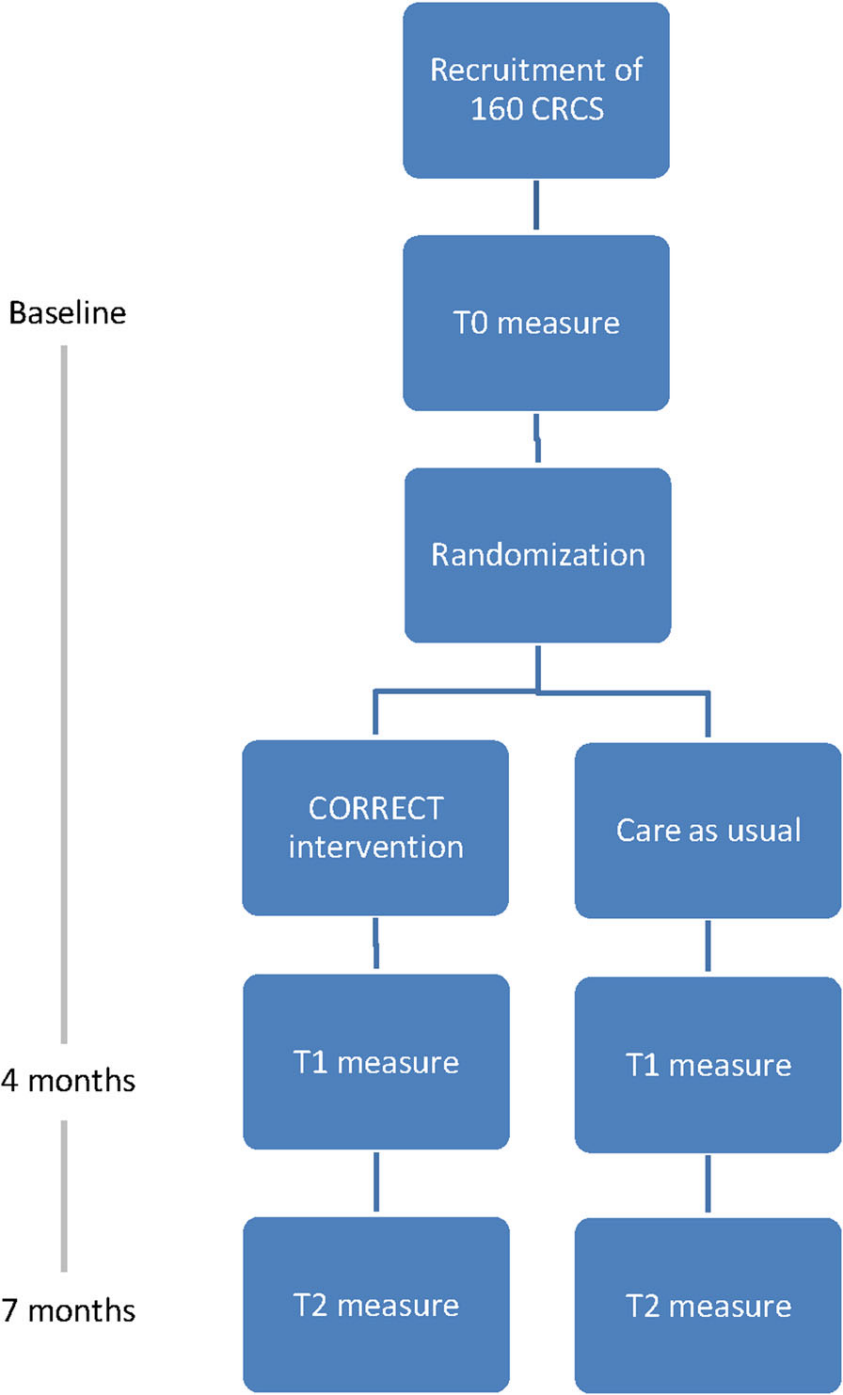
Study design of the CORRECT-study

The CORRECT study is a non-blinded, multicenter randomized controlled, two-arm trial evaluating the efficacy of the CORRECT intervention (blended CBT) compared with care as usual (CAU) in patients who have completed primary curative treatment for CRC. Participants enter the study 6 months to 5 years after completion of primary CRC treatment. After finishing the baseline assessment (T0), participants are randomly assigned to either the intervention or the CAU group. Follow-up assessments are at 4 months (T1) and 7 months (T2) after baseline. CRCS in the intervention group receive the CORRECT-intervention between T0 and T1. The CORRECT study design is summarized in Fig. 1.

### Recruitment and procedure

CRCS are approached for this study from 6 months after completion of medical treatment. Patients are recruited from the Surgery and Oncology Departments of two academic hospitals, Radboud university medical center Nijmegen (Radboudumc) and Amsterdam University Medical Centers (location VUmc), and regional hospitals. Two recruitment methods are employed. Potentially eligible patients are identified retrospectively via hospital registries. They receive mailed invitation letters from their treating physician. Alternatively, patients are prospectively recruited at routine follow-up visits. Treating nurses or physicians invite potentially eligible patients to consider participation, and provide verbal and written information about the study. In both recruitment methods, interested participants are then asked to fill out a participation form allowing contact by the researcher. Patients who do not want to consider participation are asked on voluntary basis to fill out a form containing questions about age, gender and reasons for non-participation. Following receipt of a participation form the researcher sends the patient a secure link to the digital screening questionnaire via e-mail or a hardcopy version via the mail (according to what the patient prefers). After screening, the researcher contacts the patient by phone to address further questions, confirm eligibility criteria and obtain written informed consent. During this telephone screening the researcher checks the self-perceived need for help of a patient with the question whether he/she would have problems worth talking to a psychologist about. After written informed consent, the researcher sends the patient a secure link to the digital baseline questionnaires via e-mail or a hardcopy version via the mail. Randomization occurs after receipt of a completed baseline questionnaire.

### Participant eligibility

CRCS are eligible to participate in this study if they: 1) are 18 years or older; 2) are cancer free at study entry; 3) have completed primary CRC treatment with curative intent (stage I, II or III) between 6 months and 5 years previously; 4) have high distress levels indicated by a score of ≥5 on the Distress Thermometer; 5) have basic Internet skills (e.g. possession of an email address, Internet access at home); 6) are literate in Dutch; and 7) are able to travel to the academic hospital for F2F sessions. Ineligibility criteria include: 1) diagnosis of Lynch Syndrome; 2) active psychotherapeutic treatment at study entry; and 3) inability to provide informed consent due to intellectual disability or cognitive impairment.

### Randomization

Allocation to one of the study conditions is performed using a ratio of 1:1, with blocked randomization, stratified for academic hospital (Radboudumc and VUmc), gender and diagnosis (colon and rectal cancer). Patients are randomly allocated to the CORRECT intervention or CAU with a computer randomization program developed specifically for this study. Two secretaries who are working at one central location in the Radboudumc and who are not involved in the study have access to this program and carry out randomization, thus ensuring the researcher cannot influence allocation sequence. The outcome of randomization is notified by the researcher to the patient via phone and mail.

### Sample size

Power calculation for ANCOVA analysis was conducted with G*Power 3.1.3. In order to detect a medium effect size of 0.4 (based on reviews and meta-analyses of psychosocial and cognitive behavioral interventions in cancer survivors) [25, 38–40] with an alpha of 0.05 and a power of .80, a sample size of 190 participants is calculated. To correct for the baseline assessment as covariate the sample size is multiplied with the factor (1- *r*^2^). The *r* gives the correlation between pre-intervention and post-intervention distress. Based on a meta-analysis of pre-intervention distress as a moderator in psychosocial interventions for distress in cancer patients, this is an average of 0.6 [41], resulting in a sample size of 128 patients. Taking into account a dropout rate of approximately 20% a sample of 160 patients (80 patients per condition) will be included at baseline.

### The CORRECT-intervention

#### Developmental process

Experienced clinical psychologists, cognitive behavioral therapists and researchers (JP, BT, EC, AB, LL) elaborated the website and treatment manual, including a detailed description and session checklist of each therapy session. The intervention was developed according to the theoretical framework of CBT [42] and a behavioral change model of internet interventions [43]. Further, we used the TIDieR checklist, a template for intervention description, in the developmental process [44].

The development of the CORRECT intervention consisted of different parallel stages. We started with a literature search and several expert meetings with therapists and researchers to define the core components of the intervention. In the same period, two brainstorming sessions including members of the researcher team and ICT specialists were organized to develop the structure of the website. The website was designed with technical guidance from Karify (https://www.karify.com/; Utrecht, the Netherlands), an e-health application for online information, communication and treatment in healthcare. At the start of developing the CORRECT intervention there were three existing websites which had been developed by the department of Medical Psychology in the Radboudumc [45–47]. First, was the web-based self-management intervention BREATH, designed to support the psychological adjustment of women after primary breast cancer treatment [45]. The website SWORD was developed as part of a blended therapy to manage high levels of FCR in breast, prostate and colorectal cancer survivors [46]. Following a study on psychological adjustment in the first year after diagnosis amongst Dutch CRCS (unpublished data) [48] the content of BREATH was adjusted and translated to a CRC specific self-management website. A multidisciplinary team including specialized CRC nurses, a general practitioner, psychologists, researchers and CRC patients participated in adapting this website. Finally, a think aloud study was held with five CRCS to optimize the website. This CRC specific self-management website was the basis for the CORRECT website that was further developed in accordance with the process and content of these three existing websites.

The content of the treatment manual and the website for the CORRECT-intervention was then revised by the members of the research team. The first complete version of the intervention was sent to a multidisciplinary reading committee, consisting of two nurse specialists, a surgeon, two healthcare psychologists and two CRCS. The members of the committee were asked to provide comments and suggestions in order to further improve the content of the intervention. In addition, they completed an evaluation questionnaire, which consisted of 13 items on a 5-point Likert Scale ranging from 0 to 5 (higher score indicating more positive impression) (e.g. “*What do you think of the coherence between the texts and the exercises?”*). The content of the intervention was rated with a mean score of 4.23. The content and format of the intervention were revised using feedback given by the reading committee.

In order to optimize the website, usability (i.e. the ease with which participants can use the website) of the self-management website was studied in a formal usability testing phase. Three CRCS were asked to use the website. A scenario-based think aloud procedure was employed [49]. In this procedure, the three participants were asked to verbalize their thoughts while completing tasks or going through scenarios that pose a problem. These ‘think aloud procedures’ were filmed. A researcher (LL) and a research assistant facilitated the testing sessions, documented feedback and monitored the interactions with the website. Afterwards, the videos were reviewed for content. Furthermore, participants filled out the System Usability Scale (SUS) [50, 51] and a written survey including purpose-designed open-ended (e.g. *“What do you like about the website?”*) and close-ended questions (e.g. *“Do you like the design of the website?”*). The SUS is a scale consisting of 10 items which gives a global subjective perception of the website usability. The mean total score of the SUS given by the three participants was 72.5 (range 0–100). A higher score indicates a better subjective usability perception. The website was adjusted and optimized to user-friendliness based upon feedback obtained in usability testing.

Finally, prior to commencing the RCT, the treatment manual, intervention and procedures were tested in a pilot study. It was intended that each participating therapist treats one highly-distressed CRCS so that four patients needed to be included. In total six patients were included by the screening procedure. One included patient dropped out before starting the intervention because of metastatic cancer. Another patient dropped out because of technical problems with the questionnaires and participation got too stressful. Therefore four patients started the treatment as intended. However, during the intervention two patients who scored above cut-off on the Distress Thermometer (≥5) during screening procedure appeared to have no perceived need for help. One of them dropped out of the study after three appointments with the therapist and the other stayed in the study until finishing the T1 questionnaire. Due to dropouts only three patients completed the CORRECT intervention. As a result of the pilot study, a check for self-perceived need for help with distress was added to the screening procedure. Further, a few minor changes were made in the homework exercises on the interactive website. After the pilot study, content of the CORRECT intervention and methodology were finalized.

#### Content of the intervention

The CORRECT intervention is designed to facilitate adjustment and coping and to reduce distress through changes in cognitions and behaviors. The blended therapy is tailored by the therapist in consultation with the patient to needs identified by the results of the baseline questionnaire. An online system (RadQuest software) processes the data of the baseline questionnaires and produces visual graphics into a report called the “Patient Profile Chart”, which helps interpret the results and identify problem areas [52]. Three different types of distress are targeted in the CORRECT-intervention: 1) distress due to physical consequences (gastrointestinal problems, stoma related issues, post-cancer fatigue, neuropathy, pain and sexual dysfunction), 2) anxiety and FCR, 3) depressive mood. These different types of distress are addressed in separate modules. The CORRECT-intervention is delivered over 14 weeks and consists of five individual F2F sessions of 1 h and three telephone consultations of 20 min, with simultaneous use of the self-management interactive website. The CORRECT-intervention starts with three weekly F2F sessions to discuss the Patient Profile Chart, develop the therapist-patient relationship, explain the therapy rationale, and select treatment module(s). Therapist support is gradually decreased towards the end of the intervention period, and self-management is increased through the greater use of the interactive website. This way, patients take charge of their own health and learn to cope more independently with future challenges. A similar treatment design has proven to be successful for managing FCR in breast, prostate and colorectal cancer survivors [53].

All sessions start with discussing the homework assignments which are completed on the website. In the first session the patient’s experiences of the cancer follow-up phase, current distress, unmet needs and treatment goals are discussed. The key problems and the goals of treatment are then determined. For each patient, at least one or a maximum of two modules are selected based on the initial assessment and the Patient Profile Chart. The order of the presentation is tailored to needs, with the most concerning problem addressed first. Each module has sufficient content to fill the duration of the intervention in the event that only one domain of need is identified. Patients have however free access to all the modules on the website. In the second session the therapist introduces and explains basic skills of CBT applied to the first module. The following sessions (session 3 to 7) include: psycho-education, cognitive restructuring, behavior modification and relaxation. During session 6 and 7 the therapist and patient evaluate distress reduction and discuss long-term consolidation of skills. During the final session (session 8) goal evaluation, on-going self-management, and relapse prevention are discussed. Detailed structure of the intervention can be found in Table 1.

**Table 1:** Detailed structure and timeframe of the CORRECT-intervention

Week	General introduction module		Module 1		Module 2 (optional)	General closing module	
	Session	Web-based homework	Session	Web-based homework	Web-based homework	Session	Web-based homework
0		“Preperation to first session”					
1	1: F2F	“After the first session”					
2	2: F2F			Online 1			
3			3: F2F	Online 2			
4			4: Telephone				
5				Online 3			
6			5: F2F	Online 4			
7					Online 1		
8			6: Telephone				
9				Online 5	Online 2		
10					Online 3		
11			7: Telephone				
12					Online 4		
13					Online 5		“Preperation to last session”
14						8: F2F	“Closing: how to move on?”

#### Content of the interactive self-management website

**Fig. 2:**
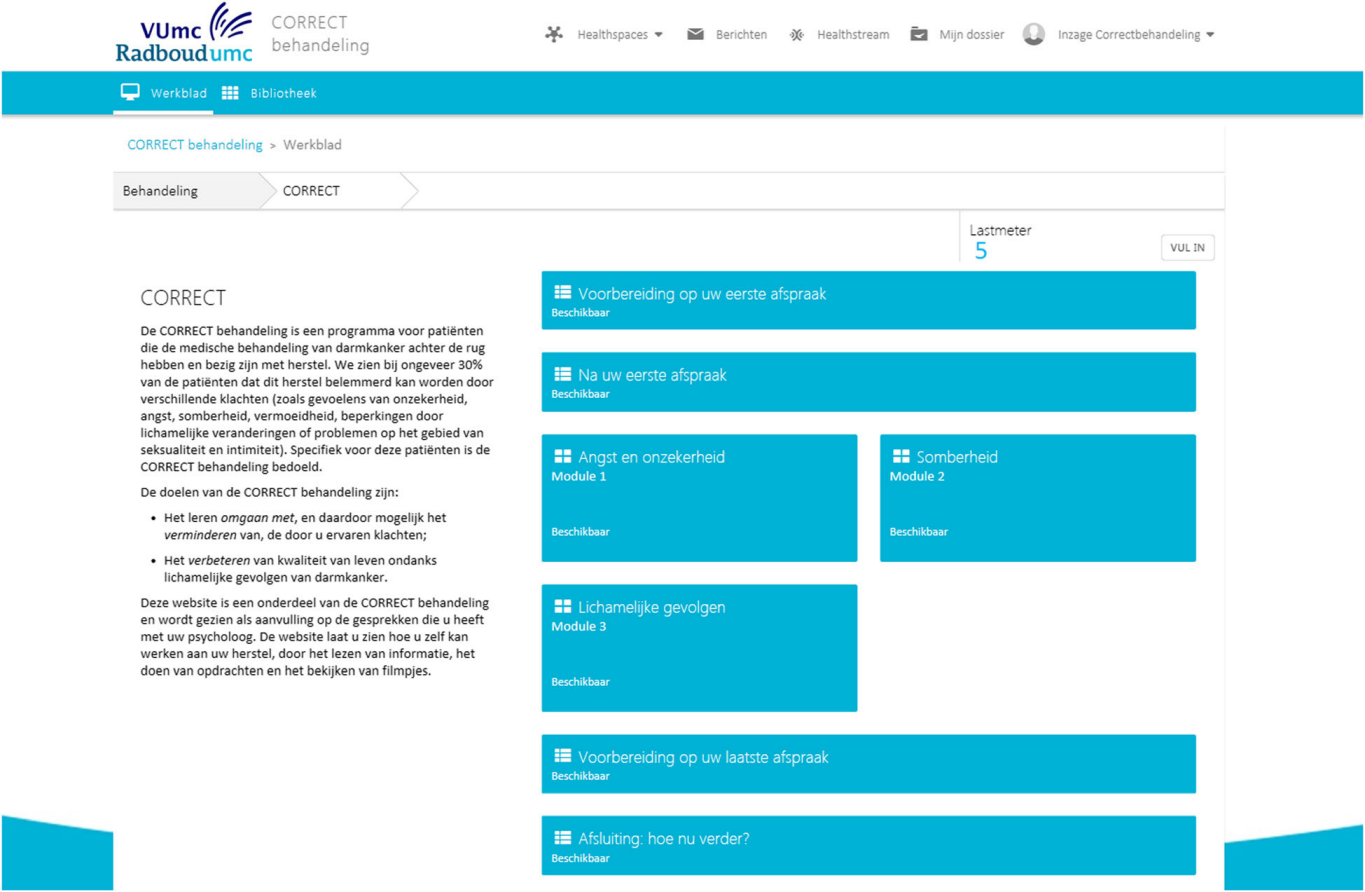
Homepage of the interactive self-management CORRECT website

The interactive self-management website is available to support the CRCS throughout the CORRECT intervention. After each F2F and telephone session, patients receive homework assignments via the website. CRCS who indicate they lack sufficient computer skills to use the website are provided with a paper workbook with identical content and a DVD or USB containing audio-visual materials. The website contains a general introduction module, the three specific modules and a general closing module (Fig. 2). The general introduction module consists of two online homework sessions including 13 exercises. These exercises focused on introducing CBT and identifying personal goals. After the general introduction module, each participant completes the chosen module(s) on the website. The three specific modules include different types of self-management activities. Each of the three specific modules has five online homework sessions with a range of 29–32 exercises, including psycho-educational scripts, assignments tasks, screening tests, audio clips, and peer videos. The peer videos were produced from edited filmed interviews between a clinical psychologist (JP) and four CRCS. Peer videos are included on the website for psycho-education and social comparison. The general closing module consists of two online homework sessions including nine exercises which are focused on goal evaluation and relapse-prevention.

#### Therapists

The therapists providing the intervention are four qualified, registered healthcare psychologists with > 10 years of experience in the field of medical psychology, psycho-oncology and/or experience in e-health therapies. All therapists completed a one-day training program and received 1 h training in use of the website. Before the trial started, three of the four therapists were able to complete treatment of one CRCS in the pilot study. All therapists used the therapist treatment manual under clinical supervision of two senior and experienced psychologists (JP, EC), of which one is a qualified CBT supervisor (JP). In both academic hospitals (Radboudumc and VUmc) two therapists are available. Therapists will be given biweekly group supervision with the two senior psychologists (JP, EC).

#### Treatment integrity

To ensure treatment integrity all therapists will use a standardized therapy manual, including therapist checklists for each session. All the F2F sessions will be audio taped. A random selection of 5% of audio-recordings will be reviewed to check fidelity to the treatment protocol.

### Control condition: care as usual

According to Dutch CRC clinical practice guidelines, survivors complete routine follow-up examinations for 5 years after completion of treatment [54]. The medical follow-up appointments are every 3–6 months during the first 2 years of follow-up, followed by (bi)-annual examinations for up to 5-years. Participants in the control condition will have access to usual care and routine follow-up. Dutch clinical practice guidelines currently stipulate that routine psychosocial screening using the Distress Thermometer should be carried out for all CRCS as part of standard follow-up care [55]. If distress is detected at screening during follow-up, CAU can be very diverse. For example, physicians or nurses may advise patients how to reduce distress or they may refer patients to their general practitioner, a social worker or a psychologist. No restrictions will be made to the CAU condition.

### Assessment

Demographic and medical information are obtained using self-report questionnaires and medical records. At three points in time (T0; baseline prior to randomization, T1; 4 months after baseline and T2; 7 months after baseline), participants are asked to complete online questionnaires using RadQuest software. Participants who do not wish to use the internet may opt for mailed paper-and-pencil questionnaires. Those who do not complete the questionnaires within 2 weeks receive a reminder from the researcher via email or by phone.

#### Screening instrument

Before inclusion in this trial, eligible patients complete a distress screening using the Distress Thermometer (DT). Patients rate their overall level of distress during the past week from 0 (no distress) to 10 (extreme distress) on a visual analog scale (the Thermometer). The DT has moderate to good internal consistency (α ranging from .60 to .90) [56, 57]. A cutoff point of ≥5 is used to identify high distress due to its optimal sensitivity, specificity and diagnostic accuracy [56, 58]. For screening in the current study, the thermometer score on the DT is used to determine eligibility. The Problem List of the DT contains 47 problems in practical, social, psychological, spiritual and physical domains; patients indicate the distress severity of each item on a 10-point scale. The Dutch version of the DT has an additional question about the wish for referral: “Would you like to talk with a professional about your problems?” [56].

#### Primary outcome

*Psychological distress* is assessed with the Brief Symptom Inventory 18-items (BSI-18) [59, 60]. The items of the BSI-18 are grouped into three subscales; anxiety, depression, and somatization. The BSI-18 gives a global severity index (GSI). The GSI is viewed as a reliable reference score sensitive to change and therefore is used to evaluate effects of psychotherapy [61]. The BSI is a valid instrument with high reliability in mixed cancer samples (α = 0.89) [59].

#### Secondary outcomes

*The perceived impact of physical consequences of colorectal cancer* is assessed with the valid and reliable Dutch version of the European Organization for Research and Treatment (EORTC) of Cancer Quality of Life Questionnaire Core 30 (QLQ-C30) [62, 63] and the 38-item colorectal cancer specific module (CR38) [64]. These questionnaires have shown good psychometric properties in survivors of cancer (α = 0.89) [62–64].

*Fatigue* is assessed using the Checklist Individual Strength (CIS) [65, 66]. The CIS is a 20-item questionnaire, designed to measure four aspects of fatigue; fatigue severity, concentration, motivation and physical activity. The CIS is a well-validated instrument [67, 68].

*Anxiety and depressed mood* is measured with the Hospital Anxiety and Depression Scale (HADS) [69, 70]. The HADS has demonstrated reliability and validity in oncology patients [71–73].

*Fear of cancer recurrence* is assessed with the Cancer Worry Scale (CWS). The CWS is able to detect dysfunctional levels of FCR [74, 75]. This scale is found to be a valid and reliable instrument in Dutch cancer survivors (α = 0.87) [75].

*Cancer-specific distress* will be assessed with a Dutch version of the Impact of Event Scale (IES). This scale measures cancer-related avoidant behaviors and intrusive cognitions [76–78]. The IES has shown good reliability (α ranging from .87 to .96) and construct validity [78].

*Self-efficacy* in dealing with distress following colorectal cancer will be assessed with the Self-Efficacy Scale (SES). This scale is previously used in measuring self-efficacy in patients with post-cancer fatigue [79, 80] and in breast cancer survivors [47].

#### Other outcome measures

*Health care utilization costs* are evaluated with a modified version of the Trimbos/iMTA questionnaire for Costs associated with Psychiatric Illness (TiC-P) [81]. Medical costs are assessed to identify health care usage (e.g. medication use/dose, visits to general practitioner or to other health care professionals). To further monitor cost-utility, the EuroQol-5D (EQ-5D) is used. The EQ-5D is a non-disease-specific instrument used to describe and value health [82, 83].

*Technical usage statistics* are obtained to evaluate website use and adherence. This is an important step in explaining how e-health interventions can cause behavior change and symptom improvement [84]. Data examined include frequency and duration of logins, type and number of opened online activities, frequency and duration of opened online activities, evaluation of online activities and number of submitted homework assignments to the therapists.

### Statistical analyses

Statistical analyses are being performed using SPSS. Key variables should be evenly distributed between conditions by randomization. To control for that, baseline characteristics are compared between participants in the intervention and CAU conditions with Chi-square (categorical variables) and ANOVA (continuous variables). Variables that are not evenly distributed are used as covariates in the analyses, along with time since end of treatment to control for the possible relationship between time since end of treatment and the level of distress. Analyses are on an intention-to-treat basis. A per-protocol analysis is amongst participants who successfully completed the intervention. ANCOVA-analysis of the change scores in the outcome variables is conducted to calculate differences between the two conditions. Exploratory sub-group analyses are conducted based on ‘time since end of treatment’, ‘age’, and ‘gender’. Caseness of the GSI is used to determine clinical significant improvement. Caseness is indicated if a T-score on the GSI scale > 62, or a T-score > 62 on two of the three clinical subscales. To analyze the difference between the proportions of patients meeting the criteria for clinically significant improvement at T1, chi-square tests are used.

### Monitoring

Data monitoring and quality assurance is conducted on a annual basis by a data monitor who is independent from the researchers and the funding body and who is employed within the Department of Medical Psychology, Radboud University Medical Centre. Annually the data monitor completes a quality monitoring document based on an interview with the researchers regarding: contact to ethical committee, study participation and design, paper and digital archives, data-analyses and controlling Informed Consent forms, Source Data Verification and Serious Adverse Events (SAE’s). SAE’s have to be reported to the ethical committee CMO Arnhem-Nijmegen by a standard procedure.

## Discussion

Approximately one third of the CRCS experience high levels of psychological distress. Due to the rising numbers of CRCS, widely accessible and evidence-based supportive care is needed to deal with this growing need. This protocol paper describes the CORRECT multicenter trial which evaluates a blended CBT intervention (CORRECT) for reducing high psychological distress in CRCS. The primary objective is to evaluate the efficacy and cost-effectiveness of the CORRECT intervention in decreasing psychological distress in CRCS. To our knowledge, this is the first blended psychological intervention with self-management elements which is specifically aimed at reducing psychological distress amongst CRCS.

CRCS are an under-served population with respect to psychosocial supportive care research. Few studies investigate psychological interventions specifically designed for CRCS. The CORRECT study addresses this gap in current research. Most studies on distress and psychosocial interventions over-represent women with breast cancer [25]. Interventions that might be proven to be effective for women with breast cancer may not be as effective for males or for survivors of other tumor types. Within CRCS samples gender may be an important mediator of the efficacy of psychological interventions. In the current study we both include male and female CRCS, which makes it possible to explore possible differences in our mixed group.

Reviews indicate that psychological interventions are most effective for patients who are pre-selected for high distress [25, 41]. In order to identify highly distressed patients, every CRCS at participating sites who scores above cut-off on the nationally mandated DT (≥ 5) will be offered inclusion in the CORRECT study. The CORRECT intervention is therefore highly-specialized and tailored care to help CRCS deal with their unique problems of survivorship. This may be a strength, however it may also be a barrier. A study by Van Scheppingen and colleagues [85] found implementing screening to be inefficient for recruiting distressed cancer survivors to a RCT. They found need for psychological services to be much lower than they anticipated before the start of the trial. In another study it was concluded that, “depending on the clinical context, screening might be more efficient if unmet needs for services are assessed rather than psychological distress” [86]. We try to obviate this issue by using a telephone protocol in which the researcher will go through the completed DT with the patient and verbally confirm there is a need for a psychological intervention. Further, we expect our retrospective recruitment method to overcome this issue by first asking if a patient is willing to participate in the trial and then screening the patient for high distress level. An additional strength of this trial is that it is conducted using a rigorous methodology and in accordance with CONSORT guidelines. Patient recruitment is conducted in different hospitals (both regional and academic hospital settings) in two regions of the Netherlands, which may enhance its generalizability. Furthermore, it is delivered by experienced therapists who are working in clinical practice and not therapists specially recruited and trained for therapy delivery in academic centers. This will facilitate implementation should the therapy be proven effective.

The CORRECT intervention is developed in close collaboration with patients from the participating hospitals and patient representatives from patient organizations. CRCS have been involved in different phases of the research and intervention development. A pilot study has demonstrated that the screening procedure is feasible and acceptable to CRCS. Patient participation in the CORRECT study helps to ensure that the intervention is provided in a manner consistent with patient needs and preferences. Further, we aimed to use the person-based approach to ground our intervention design as intended: “in a rigorous, in-depth understanding of the psychosocial context of the people who will use the intervention” [87]. Taking the needs and experiential knowledge of patients into account is considered to result in the improvement of individual health care [88]. Patient participation in the intervention development will help ensure the intervention is provided in a manner consistent with patient needs and preferences. If proven effective, this will increase the likelihood of high uptake when implemented in routine care.

A secondary objective of the CORRECT study is to investigate the use of online activities and how website usage is associated with distress reduction, in accordance with usage evaluation in a previous study of online self-management intervention for breast cancer survivors [84]. Usage evaluations are relatively new and growing area of interest in online intervention research. Analysis of usage data provides information on which patient subgroups experience the greatest benefits. This facilitates knowledge about personalizing psychosocial interventions for CRCS and further a growing body of research on the relationship between e-health interventions and psychological and behavioral change.

In conclusion, the CORRECT intervention is a promising method of reducing psychological distress, improving QoL and enhancing personalized supportive care for CRCS. Should this trial prove its efficacy, the ultimate goal will be to implement and disseminate the CORRECT intervention nationally and internationally.

## Abbreviations

CAU: Care as usual
CBT: Cognitive behavioral therapy
CMO: Medical ethical committee
CRC: Colorectal cancer
CRCS: Colorectal cancer survivor(s)
F2F: Face-to-face
FCR: Fear of cancer recurrence
QoL: Quality of life
Radboudumc: Radboud University Medical Center
RCT: Randomized controlled trial
VUmc: VU University Medical Center
